# PRAS40 Is an Integral Regulatory Component of Erythropoietin mTOR Signaling and Cytoprotection

**DOI:** 10.1371/journal.pone.0045456

**Published:** 2012-09-18

**Authors:** Zhao Zhong Chong, Yan Chen Shang, Shaohui Wang, Kenneth Maiese

**Affiliations:** 1 Laboratory of Cellular and Molecular Signaling, Newark, New Jersey, United States of America; 2 Cancer Institute of New Jersey, New Brunswick, New Jersey, United States of America; 3 New Jersey Health Sciences University, Newark, New Jersey, United States of America; Universidad de Castilla-La Mancha, Spain

## Abstract

Emerging strategies that center upon the mammalian target of rapamycin (mTOR) signaling for neurodegenerative disorders may bring effective treatment for a number of difficult disease entities. Here we show that erythropoietin (EPO), a novel agent for nervous system disorders, prevents apoptotic SH-SY5Y cell injury in an oxidative stress model of oxygen-glucose deprivation through phosphatidylinositol-3-kinase (PI 3-K)/protein kinase B (Akt) dependent activation of mTOR signaling and phosphorylation of the downstream pathways of p70 ribosomal S6 kinase (p70S6K), eukaryotic initiation factor 4E-binding protein 1 (4EBP1), and proline rich Akt substrate 40 kDa (PRAS40). PRAS40 is an important regulatory component either alone or in conjunction with EPO signal transduction that can determine cell survival through apoptotic caspase 3 activation. EPO and the PI 3-K/Akt pathways control cell survival and mTOR activity through the inhibitory post-translational phosphorylation of PRAS40 that leads to subcellular binding of PRAS40 to the cytoplasmic docking protein 14-3-3. However, modulation and phosphorylation of PRAS40 is independent of other protective pathways of EPO that involve extracellular signal related kinase (ERK 1/2) and signal transducer and activator of transcription (STAT5). Our studies highlight EPO and PRAS40 signaling in the mTOR pathway as potential therapeutic strategies for development against degenerative disorders that lead to cell demise.

## Introduction

Neurodegenerative disease leads to either severe disability or death for a significant proportion of the world's population. For example, in regards to cognitive disease, it is estimated that greater than twenty-four million people are afflicted with Alzheimer's disease, pre-senile dementia, and associated disease that involve memory loss [1], [2], [3]. Although multiple factors may contribute to the onset and progression of neurodegenerative disease, oxidative stress is considered to be an important component in neurodegenerative disorders. Oxidative stress can lead to cognitive disorders [4], [5], [6], movement disorders [5], [7], [8], and neurovascular complications associated with metabolic disease [9], [10], [11].

Given that effective treatments for the majority of neurodegenerative disorders do not exist, new strategies that can offer protection in the nervous system during oxidative stress are of great interest [12], [13]. In particular, erythropoietin (EPO) represents a novel therapy that may provide robust protection for both neuronal and non-neuronal cells in the nervous system. EPO prevents neuronal cell injury [14], [15], [16], [17], [18], [19], [20], maintains vascular integrity [21], [22], [23], [24], and modulates inflammatory cell activation [25], [26], [27], [28]. EPO promotes cellular survival through the phosphatidylinositol-3-kinase (PI 3-K) and protein kinase B (Akt) pathways [29], [30], [31], [32]. More recent studies have demonstrated that EPO also relies upon mammalian target of rapamycin (mTOR) signaling to modulate inflammatory cell survival [27], [33], osteoblastogenesis, and osteoclastogenesis [34].

In a number of scenarios, mTOR activation may be necessary to prevent apoptotic neuronal cell death during oxidative stress. Cell death following exposure to oxidative stress in dopaminergic neurons can be prevented during application of agents that increase mTOR activity [35]. In contrast, loss of mTOR activity during oxidative stress leads to apoptotic neuronal death [36] and injury in non-neuronal inflammatory cells [33], [37]. One of the central pathways that can control mTOR signaling is the proline rich Akt substrate 40 kDa (PRAS40). Through mTOR Complex 1 (mTORC1), PRAS40 prevents mTOR activity and inhibits the binding of the downstream mTOR proteins p70 ribosomal S6 kinase (p70S6K) and the eukaryotic initiation factor 4E-binding protein 1 (4EBP1) to Raptor [38], [39], [40]. PRAS40 activity is inhibited during post-translational phosphorylation [41] and this has been associated with increased cell survival [42], [43], [44]. We therefore examined if PRAS40 was a critical regulatory pathway for EPO to foster neuroprotection during oxidative stress. We show that in a model of oxygen-glucose deprivation (OGD) that can lead to oxidative stress [45], [46], EPO activates mTOR signaling through PI 3-K/Akt pathways to phosphorylate p70S6K and 4EBP1 that is necessary for protection in differentiated SH-SY5Y cells. EPO controls cell survival and mTOR activity through the post-translational phosphorylation of PRAS40 and the binding of PRAS40 to 14-3-3 protein. Furthermore, inhibition of PRAS40 is an integral cytoprotective component of EPO that can increase cell survival and limit apoptotic caspase 3 activity independent of other protective pathways of EPO that involve extracellular signal related kinase (ERK 1/2) and signal transducer and activator of transcription (STAT5). Our work highlights PRAS40 in the cytoprotective pathways of EPO as a potential target for novel therapeutic strategies directed against degenerative disorders.

## Materials and Methods

### Human neuroblastoma SH-SY5Y cell culture and differentiation

Per our prior protocols [47], [48], human adrenergic neuroblastoma SH-SY5Y cells were purchased from ATCC (American Type Culture Collection) and maintained in regular Dulbecco's modified Eagle medium (DMEM) (Life Technologies Corp, Carlsbad, CA), supplemented with 10% heat-inactivated fetal bovine serum, 1 mM pyruvate, 1.5 g/L sodium bicarbonate, 100 IU/ml penicillin, 100 µg/ml streptomycin at 37°C in 95%/5% (v/v) mixture of humidified atmospheric air and CO_2_. Cell suspension was prepared at a density of 3–4×10^4^ (24 well plate) or 1–1.5×10^5^ (35 mm^2^ Petri dish). When confluent at 50–60%, cells were differentiated by MEM growth medium containing 10 μM all-trans retinoic acid (RA) (Sigma, St. Louis, MO) for 48 h. Experiments were initiated until cells grew to 60% – 70% confluence between passages 4–10 after differentiation.

### Experimental treatments

Per our prior experimental protocols, oxygen-glucose deprivation (OGD) in SH-SY5Y cells was performed by replacing the media of the cultures in 35 mm^2^ dishes with cells of 60–70% confluence with glucose-free Hank's balanced salt solution (HBSS) containing 116 mmol/l NaCl, 5.4 mmol/l KCl, 0.8 mmol/l MgSO_4_, 1 mmol/l NaH_2_PO_4_, 0.9 mmol/l CaCl_2_, and 10 mg/l phenol red (pH 7.4). SH-SY5Y cultures were then placed into a Bactron II anaerobic glove box (Sheldon manufacturing, Inc, Cornelius, OR) and were maintained in an anoxic environment (95% N_2_ and 5% CO_2_) at 37°C for 6 hours. Following this period, the cultures were removed from the anoxic chamber and the glucose-free HBSS was replaced with media containing Dulbecco's modified Eagle medium (DMEM) (Life Technologies Corp, Carlsbad, CA), supplemented with 10% heat-inactivated fetal bovine serum, 1 mM pyruvate, 1.5 g/L sodium bicarbonate, 100 IU/ml penicillin, and 100 µg/ml streptomycin and maintained at 37°C in 95%/5% (v/v) mixture of humidified atmospheric air and CO_2_. For treatments applied prior to OGD, erythropoietin (Sigma, St. Louis, MO), the phosphatidylinositol-3-kinase (PI 3-K) inhibitors wortmannin (0.5 μM, EMD Biochemicals Inc, Gibbstown, NJ) and LY294002 (10 μM, Sigma, St Louis, MO), extracellular signal-regulated kinase (ERK) inhibitor 3-(2-aminoethyl)-5-((4-ethoxyphenyl) methylene)-2,4-thiazolidinedione (ERK-1) (100 μM) (EMD Biochemicals Inc, Gibbstown, NJ), HCl (ERK-I) (EMD Biochemicals Inc, Gibbstown, NJ), and signal transducer and activator of transcription- 5 (STAT5) inhibitor N′-((4-Oxo-4H-chromen-3-yl)methylene) nicotinohydrazide (STAT5-I) (100 μM) (EMD Biochemicals Inc, Gibbstown, NJ) were each administered directly to the cultures 1 hour prior to OGD and treatments were continuous.

### Assessment of cell survival

SH-SY5Y cell injury was determined by bright field microscopy using a 0.4% trypan blue dye exclusion method 24 hours following treatment with OGD per our previous protocols [22], [49]. For each experimental condition, 8×35 mm^2^ dishes were used, and for each dish, the mean survival was determined by counting eight randomly selected non-overlapping fields with each containing approximately 20 cells (viable + non-viable). Each experiment was replicated 6 times with different cultures.

### Assessment of DNA Fragmentation

Genomic DNA fragmentation was determined by the terminal deoxynucleotidyl transferase nick end labeling (TUNEL) assay [47], [50], [51]. Briefly, SH-SY5Y cells were fixed in 4% paraformaldehyde/0.2% picric acid/0.05% glutaraldehyde and the 3′-hydroxy ends of cut DNA were labeled with biotinylated dUTP using the enzyme terminal deoxytransferase (Promega, Madison, WI) followed by streptavidin-peroxidase and visualized with 3,3′-diaminobenzidine (Vector Laboratories, Burlingame, CA).

### Expression of mTOR, p70S6K, Akt1, STAT5, 4EBP1, PRAS40, ERK1/2, and caspase 3 with relevant phosphorylated moieties

Cells were homogenized and following protein determination, each sample (25–50 μg/lane) was then subjected to 7.5% (p-mTOR, mTOR, p-p70S6K, p70S6K, p-Akt1, Akt1, and p-STAT5, STAT5) or 12.5% (p-4EBP1, 4EBP1, PRAS40, p-PRAS40, ERK 1/2, p-ERK 1/2, and caspase 3) SDS-polyacrylamide gel electrophoresis separation. After blocking for 1 hour at room temperature with 5% skim milk, the membranes were incubated overnight at 4°C with a rabbit antibody against (p-  =  phosphorylated) p-mTOR (Ser ^2448^, 1∶1000), mTOR (1∶1000), p-p70S6K (Thr^389^, 1∶1000), p70S6K (1∶1000), p-Akt1 (Ser^473^, 1∶1000), Akt1 (1∶1000), p-STAT5 (Tyr^694^, 1∶1000), STAT5 (1∶1000), p-4EBP1 (Ser ^65^/Thr^70^, 1∶1000), 4EBP1 (1∶1000), PRAS40 (1∶1000), p-PRAS40 (Thr^246^, 1∶1000), p-ERK 1/2 (Thr^202^/Tyr^204^), and cleaved caspase 3 (1∶1000). All antibodies were obtained from Cell Signaling, Beverly, MA. Following incubation, the membranes were incubated with a horseradish peroxidase (HRP) conjugated secondary antibody goat anti-rabbit IgG (goat anti-rabbit IgG, 1∶5000, Thermo Scientific, Rockford, IL). The antibody-reactive bands were revealed by chemiluminescence (Amersham Pharmacia Biotech, Piscataway, NJ) and band density was performed using the public domain NIH Image program (developed at the U.S. National Institutes of Health and available at http://rsb.info.nih.gov/nih-image/).

### *In vitro* assay of phosphorylation of mTOR and PRAS40

Recombinant human mTOR (EMD Biochemicals Inc, Gibbstown, NJ) or PRAS40 protein (Enzo Life Sciences, Plymouth Meeting, PA) 1 μg was incubated with 10 ng/ml EPO for 30 min at 30°C under continuous agitation in 30 μl kinase buffer containing 200 μM ATP (Cell Signaling Technology, Beverly, MA). Samples were analyzed by Western blot analysis using SDS-polyacrylamide gel and rabbit antibody against p-mTOR (Ser ^2448^) or p-PRAS40 (Thr^246^) (Cell Signaling Technology, Beverly, MA).

### Gene silencing of *Akt1* and *PRAS40* with small interfering RNA (siRNA)

Cells were plated into 35 mm dishes or 24-well plates. To silence *Akt1* and *PRAS40* gene expression, commercial reagents using the SMARTpool Akt1 siRNA kit (Millipore, Billerica, MA) and siRNA pool for PRAS40 (Santa Cruz, Santa Cruz, CA) were used respectively. Transfection of siRNA duplexes was performed with Lipofectamine 2000 reagent according to manufacturer guidelines (Life Technologies, Carlsbad, CA). Experimental assays were performed 72 hours post-transfection. For each siRNA assay, scrambled siRNA was used as control.

### Immunoprecipitation of 14-3-3, mTOR or PRAS40

Cell lysates of total protein (200 μg) were incubated with primary antibody against protein 14-3-3 (1∶100, Santa Cruz Biotech, Santa Cruz, CA) or mTOR (1∶100, Cell Signaling Technology, Beverly, MA) overnight at 4°C. The complexes were collected with protein A/G-agarose beads, centrifuged, and then prepared for 14-3-3, mTOR, PRAS40, and p-PRAS40 western analysis.

### Statistical analysis

For each experiment, the mean and standard deviation (SD) was determined. Statistical differences among groups were assessed by means of analysis of variance (ANOVA) with the post-hoc Dunnett's test. Statistical significance was considered at P<0.05.

## Results

### EPO prevents cellular injury and apoptotic genomic DNA degradation during oxygen glucose deprivation (OGD)

**Figure 1:**
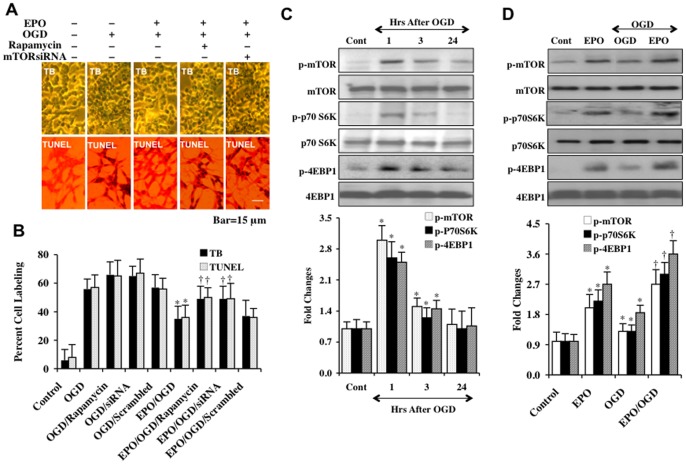
EPO promotes cellular protection through mTOR activation and phosphorylation of p70S6K and 4EBP1. (**A**) EPO (10 ng/ml) was applied to SH-SY5Y cultures 1 hour prior to a 6 hour-period of OGD and cell survival and DNA fragmentation was determined 24 hours later through trypan blue dye exclusion (TB) and TUNEL assay. Representative images show that EPO significantly reduces trypan blue and TUNEL staining following OGD. However, application of the mTOR inhibitor rapamycin (50 nM) or transfection of mTOR siRNA prior to EPO administration resulted in the loss of EPO cytoprotection during OGD. (**B**) Quantitative results illustrate that EPO (10 ng/ml) significantly reduces percent cell staining of trypan blue (TB) and TUNEL in SH-SY5Y cells 24 hours following OGD. Rapamycin (50 nM) or mTOR siRNA transfection prior to OGD attenuated the ability of EPO to offer cytoprotection during OGD leading to an increase in the percent staining of trypan blue and TUNEL. Transfection of scrambled siRNA did not alter EPO cytoprotection or cell injury when compared to treatment with EPO or OGD alone (*P<0.01 vs. OGD; ^†^ *P*<0.01 vs. EPO/OGD). For **B**, each data point represents the mean and SD from 6 experiments. (**C**) Western blot was performed for phosphorylated (p-) mTOR (p-mTOR, Ser^2448^), phosphorylated (p-)-p70S6K (p-p70S6K, Thr^389^), phosphorylated (p-)-4EBP1 (p-4EBP1, Ser^65^/Thr^70^) in SH-SY5Y cells at 1, 3, or 24 hours (Hrs) following a 6 hour period of OGD exposure. OGD resulted in a transient increase in the expression of p-mTOR, p-p70S6K, and p-4EBP1 at 1 and 3 hours (*P<0.01 vs. Control). (**D**) EPO (10 ng/ml) administration to SH-SY5Y cells significantly increased the expression of p-mTOR, p-p70S6K, and p-4EBP1 3 hours later. EPO (10 ng/ml) applied to SH-SY5Y cells 1 hour prior to a 6 hour period of OGD significantly increased the expression of p-mTOR, p-p70S6K, and p-4EBP1 3 hours following OGD when compared to OGD treated alone (*P<0.01 vs. untreated control; ^†^ *P*<0.01 vs. OGD treated alone). For **C** and **D**, Cont =  Control and each data point represents the mean and SD from 3 experiments.

EPO (10 ng/ml) was administered to cell cultures 1 hour prior to a 6 hour period of OGD and cell injury was determined 24 hours later through trypan blue dye exclusion method and TUNEL assay. This concentration of EPO was chosen since it previously was shown to provide significant cytoprotection in neuronal cells and vascular cells [21], [22], [50], [52]. In Figures 1A and 1B, untreated SH-SY5Y cells were not significantly stained with trypan blue and TUNEL, but exposure to OGD resulted in significant trypan blue staining and nuclear DNA damage 24 hours following OGD exposure in neurons. In contrast, EPO (10 ng/ml) significantly reduced trypan blue staining and nuclear DNA degradation.

### EPO provides cellular protection through mTOR and its signaling pathways

Application of rapamycin (50 nM) or transfection with mTOR siRNA during EPO (10 ng/ml) exposure increased cell injury and DNA fragmentation following OGD when compared to OGD exposure alone (Figures 1A and 1B). Protection by EPO was significantly reduced during blockade of mTOR and its signaling pathways, suggesting that EPO relies upon mTOR to offer cellular protection during oxidant stress. As a control, non-specific scrambled siRNA during EPO treatment did not alter survival or DNA fragmentation when compared to EPO treatment and OGD exposure alone.

### Treatment with EPO leads to mTOR activation and phosphorylation of p70S6K and 4EBP1

Given that inhibition of mTOR can block cytoprotection by EPO during OGD, we investigated whether EPO can activate mTOR and control the activity of its downstream targets p70S6K and 4EBP1. The carboxy-terminal (C-terminal) kinase domain of mTOR consists of a conserved sequence with homology to the catalytic domain of phosphoinositide 3 –kinase (PI 3-K) with phosphorylation sites of mTOR for its activation [41], [53] that include serine^2448^ [54]. Serine^2448^ is an important target for both Akt and p70S6K [54], [55]. mTOR phosphorylates and activates p70S6K at threonine^389^ that serves as a marker of mTOR activity [56]. 4EBP1 is phosphorylated by mTOR at serine^65^ and threonine^70^ [57]. Phosphorylation of 4EBP1 leads to the dissociation of 4EBP1 from eukaryotic translation initiation factor 4 epsilon (eIF4E) to allow the eukaryotic translation initiation factor 4 gamma (eIF4G) to begin mRNA translation [58], [59].

We assessed the expression of phosphorylated mTOR (p-mTOR, Ser^2448^, active form) and phosphorylated forms of its downstream targets p-p70S6K and p-4EBP1 (p-p70S6K, Thr^389^; p-4EBP1, Ser^65^/Thr^70^) during OGD exposure. In Figure 1C, the expression of p-mTOR, p-p70S6K, and p-4EBP1 was significantly increased at 1 and 3 hours following OGD exposure, but returned to the level of untreated controls within 24 hours following OGD exposure. In the next series of studies, treatment with EPO (10 ng/ml) alone significantly increased the expression of p-mTOR, p-p70S6K, and p-4EBP1 within 3 hours after EPO exposure (Figure 1D).

### EPO employs mTOR to phosphorylate p70S6K and 4EBP1

**Figure 2:**
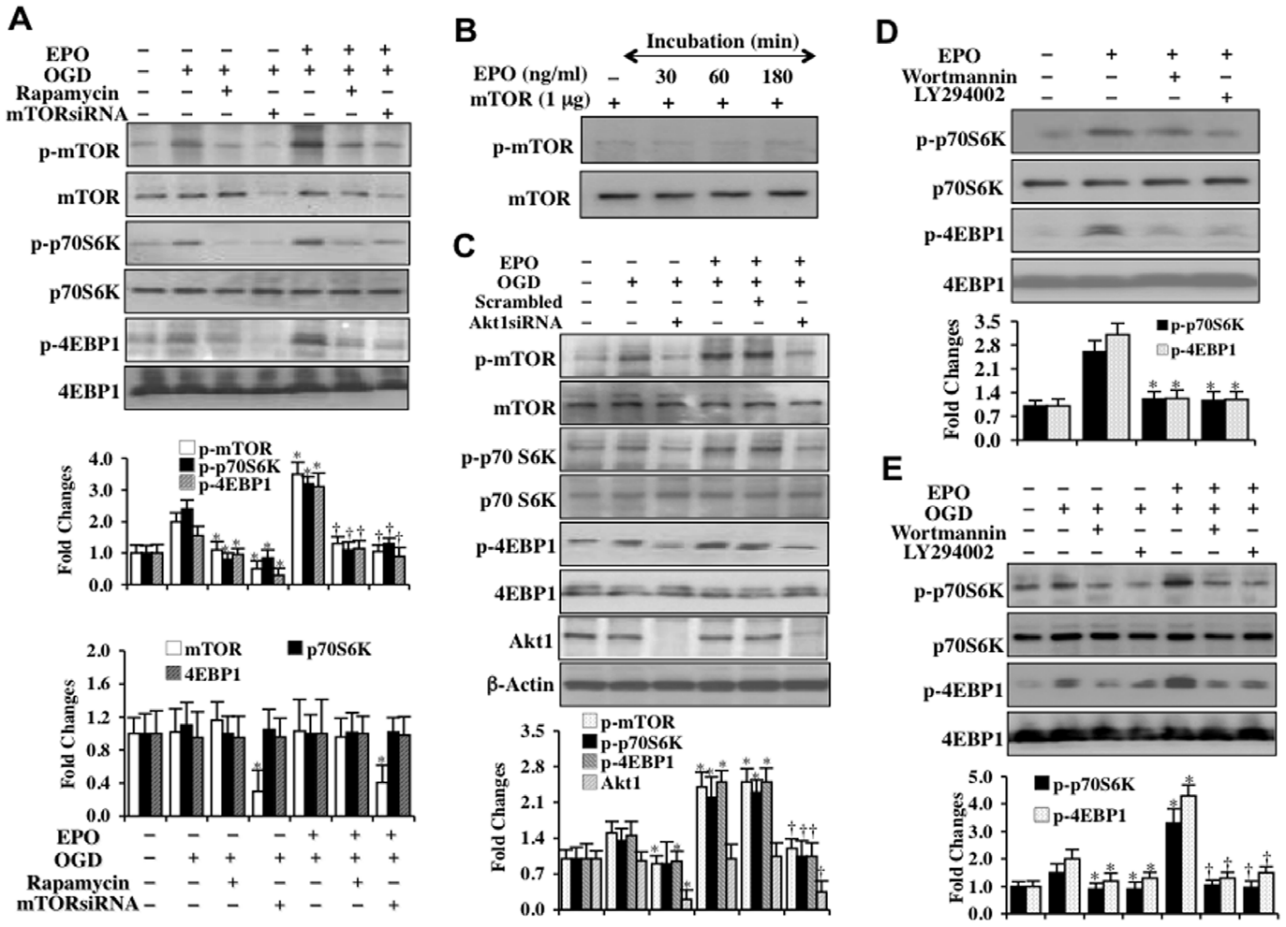
EPO phosphorylation of mTOR, p70S6K, and 4EBP1 is dependent upon PI 3-K and Akt. (**A**) Rapamycin (50 nM) administration or mTOR siRNA transfection during OGD exposure alone or treatment with EPO (10 ng/ml) applied 1 hour prior to OGD prevented the phosphorylation (p-) of p-mTOR, p-p70S6K, and p-4EBP1 3 hours following OGD exposure. Transfection with mTOR siRNA also significantly limited the expression of total mTOR (*P<0.01 vs. OGD treated alone; ^†^ *P*<0.01 vs. EPO/OGD). (**B**) EPO (10 ng/ml) was incubated with recombinant mTOR protein (1 μg) for 30, 60, and 180 min. No significant expression of phosphorylated (p-) mTOR was detected. (**C**) Akt1 siRNA transfection in SH-SY5Y cells prior to OGD or prior to treatment with EPO (10 ng/ml) applied 1 hour prior to OGD significantly reduced the expression of total Akt1, phosphorylated (p-) p-mTOR, p-p70S6K, and p-4EBP1 3 hours following OGD (*P<0.01 vs. OGD treated alone; ^†^ *P*<0.01 vs. EPO/OGD). (**D**) EPO (10 ng/ml) or EPO combined with wortmannin (500 nM) or LY294002 (20 μM) was applied to SH-SY5Y cells. Western blot analysis was performed to detect the expression of phosphorylated (p-) p-p70S6K and p-4EBP1 3 hours later. EPO phosphorylation (p-) of p-p70S6K and p-4EBP1 was blocked by the PI 3-K inhibitors wortmannin or LY294002 (*P<0.01 vs. EPO treated alone). (**E**) EPO (10 ng/ml) or EPO combined with wortmannin (500 nM) or LY294002 (20 μM) was applied to SH-SY5Y cells 1 hour prior to OGD and western blot was performed to detect the expression phosphorylated (p-) p-p70S6K and p-4EBP1 3 hours following OGD. EPO phosphorylation (p-) of p-p70S6K and p-4EBP1 during OGD was blocked by the PI 3-K inhibitors wortmannin or LY294002 (*P<0.01 vs. OGD treated alone; ^†^ *P*<0.01 vs. EPO/OGD). In all cases above, each data point represents the mean and SD from 3 experiments.

EPO (10 ng/ml) given 1 hour prior to OGD also significantly increased the expression of phosphorylated p-mTOR, p-p70S6K, and p-4EBP1 3 hours following OGD exposure, illustrating that EPO activates mTOR and p70S6K through phosphorylation but inhibits 4EBP1 activation through phosphorylation. The phosphorylation of mTOR, p70S6K, and 4EBP1 during either exposure to OGD alone or exposure to EPO and OGD were prevented during rapamycin administration or the transfection with mTOR siRNA (Figure 2A). During loss of mTOR activity or the gene silencing of mTOR, phosphorylation of mTOR, p70S6K, and 4EBP1 is minimal or absent during administration of EPO with OGD, demonstrating that EPO relies upon mTOR to phosphorylate p70S6K and 4EBP1 (Figure 2A). Furthermore, transfection with mTOR siRNA is confirmed by western analysis to prevent the expression of phosphorylated p-mTOR and total mTOR (Figure 2A).

### Phosphorylation of mTOR, p70S6K, and 4EBP1 by EPO are dependent upon PI 3-K and Akt pathways

Under some conditions, pathways for cellular proliferation and survival require the involvement of mTOR and phosphoinositide 3 –kinase (PI 3-K)/Akt pathways [60]. Since EPO employs mTOR to modulate the phosphorylation of p-p70S6K and p-4EBP1 (Figure 2A), we assessed whether EPO also relies upon the PI 3-K/Akt pathway to phosphorylate p-p70S6K and p-4EBP1. We initially examined whether EPO could directly phosphorylate mTOR. In Figure 2B, recombinant mTOR protein was incubated with EPO (10 ng/ml) for 30, 60, and 180 minutes in kinase assay buffer with western analysis subsequently performed. No significant expression of p-mTOR was detected over a 3 hour period of incubation, illustrating that EPO does not directly phosphorylate and activate mTOR. In Figure 2C, phosphorylation of p-mTOR by EPO (10 ng/ml) during OGD exposure was prevented with application of the PI 3-K inhibitors wortmannin (500 nM) or LY294002 (20 μM), illustrating that EPO phosphorylation of mTOR was dependent upon activation of the PI 3-K pathway. The inhibitor wortmannin forms a covalent link with the lysine residue of PI 3-K [61] and the inhibitor LY294002 (20 μM) reversibly competes for ATP binding to inhibit the PI 3-K pathway [62]. In addition, transfection of Akt1 siRNA also prevents EPO (10 ng/ml) phosphorylation of p-mTOR, p-p70S6K, and p-4EBP1 during OGD exposure (Figure 2C), demonstrating that Akt1 also is required for EPO to phosphorylate and activate mTOR as well as phosphorylate p-p70S6K and p-4EBP1. In Figures 2D and 2E, EPO (10 ng/ml) significantly increased the expression of phosphorylated p-p70S6K and p-4EBP1 alone and during exposure to OGD (Figures 2D and 2E). However, phosphorylation of p-p70S6K and p-4EBP1 by EPO alone or during OGD exposure was blocked during application of the PI 3-K inhibitors wortmannin (500 nM) and or LY294002 (20 μM), suggesting that EPO also employs the PI 3-K pathway to phosphorylate p70S6K and 4EBP1.

### Loss of PRAS40 prevents cell injury, improves EPO cytoprotection, and increases phosphorylation of mTOR, p70S6K, and 4EBP1

**Figure 3:**
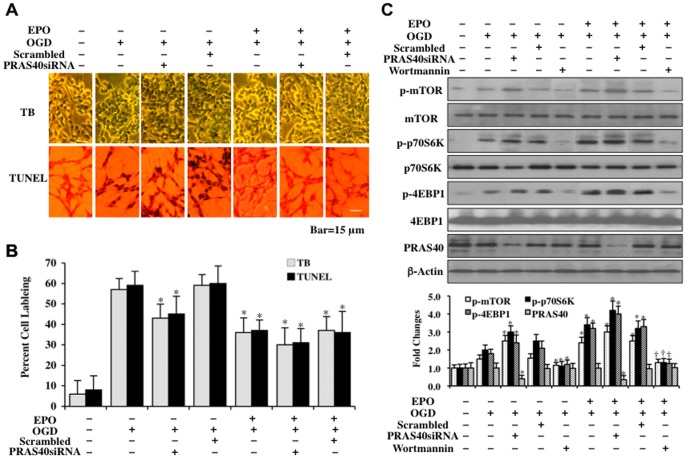
Loss of PRAS40 prevents cell demise, improves EPO cytoprotection, and increases phosphorylation of mTOR, p70S6K, and 4EBP1. (**A**) Representative images of trypan blue (TB) dye staining and TUNEL assay performed 24 hours following OGD exposure in SH-SY5Y cells show that PRAS40 siRNA transfection prior to OGD significantly reduced trypan blue and TUNEL staining and mildly improved cytoprotection by EPO (10 ng/ml) applied 1 hour prior to OGD. (**B**) Quantitative results of cell survival assessed by trypan blue dye exclusion (TB) and DNA fragmentation by TUNEL assay demonstrate that PRAS40 siRNA transfection during OGD alone or during EPO (10 ng/ml) with OGD significantly decreased percent staining of trypan blue and TUNEL 24 hours following OGD (*P<0.01 *vs.* OGD treated alone). Scrambled siRNA transfection did not alter cell injury during OGD exposure or EPO treatment with OGD. (**C and D**) PRAS40 siRNA was transfected into SH-SY5Y cells prior to OGD and western blot analysis for phosphorylated (p-) p-mTOR, p-p70S6K, p-4EBP1, and total PRAS40 was performed in cell extracts at 3 hour following OGD. EPO (10 ng/ml) applied 1 hour prior to OGD significantly increased the expression of p-mTOR, p-p70S6K and p-4EBP1. Transfection with PRAS40 siRNA increased, although the increase is not statistically significant, the expression of p-mTOR, p-p70S6K and p-4EBP1 during OGD alone and during EPO (10 ng/ml) with OGD exposure. Transfection with PRAS40 siRNA significantly limited the expression of total PRAS40. Scrambled siRNA did not alter expression of p-mTOR, p-p70S6K. p-4EBP1, total PRAS40 during OGD alone and during EPO (10 ng/ml) with OGD exposure (*P<0.01 vs. OGD; ^†^ *P*<0.01 vs. EPO/OGD). In all cases above, each data point represents the mean and SD from 3 experiments.

PRAS40 can inhibit mTOR signaling by associating with Raptor [39], [40] and prevent p70S6K and 4EBP1 binding to Raptor [38], [40]. We therefore examined whether loss of PRAS40 could alter cell survival and apoptotic DNA degradation following OGD exposure and EPO administration. Cell survival was assessed with trypan dye blue exclusion and DNA degradation with TUNEL 24 hours after OGD exposure. In Figures 3A and 3B, transfection with PRAS40 siRNA during OGD exposure significantly decreased trypan blue staining and TUNEL staining, illustrating that loss of PRAS40 is protective during OGD exposure. In addition, gene silencing of *PRAS40* during EPO (10 ng/ml) administration and OGD exposure improved cell survival and limited genomic DNA degradation to a greater extent than EPO alone (Figures 3A and 3B), suggesting that EPO employs inhibition of PRAS40 to some degree to offer cytoprotection during OGD exposure. As a control, non-specific scrambled siRNA during EPO treatment did not alter survival or DNA fragmentation when compared to EPO treatment and OGD exposure alone.

In Figure 3C, we examined the effects of *PRAS40* gene silencing upon the expression of phosphorylated p-mTOR, p-p70S6K, and p-4EBP1 during OGD exposure and exposure to EPO and OGD. Transfection with PRAS40 siRNA significantly limited the expression of PRAS40 protein and increased the phosphorylation of p-mTOR and its activity as suggested by increased phosphorylation of p-p70S6K and p-4EBP1 (Figure 3C). Loss of PRAS40 with siRNA transfection also increased the phosphorylation of p-mTOR, p-p70S6K, and p-4EBP1 during EPO (10 ng/ml) administration with OGD, suggesting that EPO phosphorylation of mTOR, p70S6K, and 4EBP1 can be fostered by the inhibition or loss of PRAS40 (Figure 3C). As a control, non-specific scrambled siRNA did not alter mTOR, p70S6K, and 4EBP1 phosphorylation.

### EPO maintains PRAS40 phosphorylation through PI 3-K and Akt during OGD exposure

**Figure 4:**
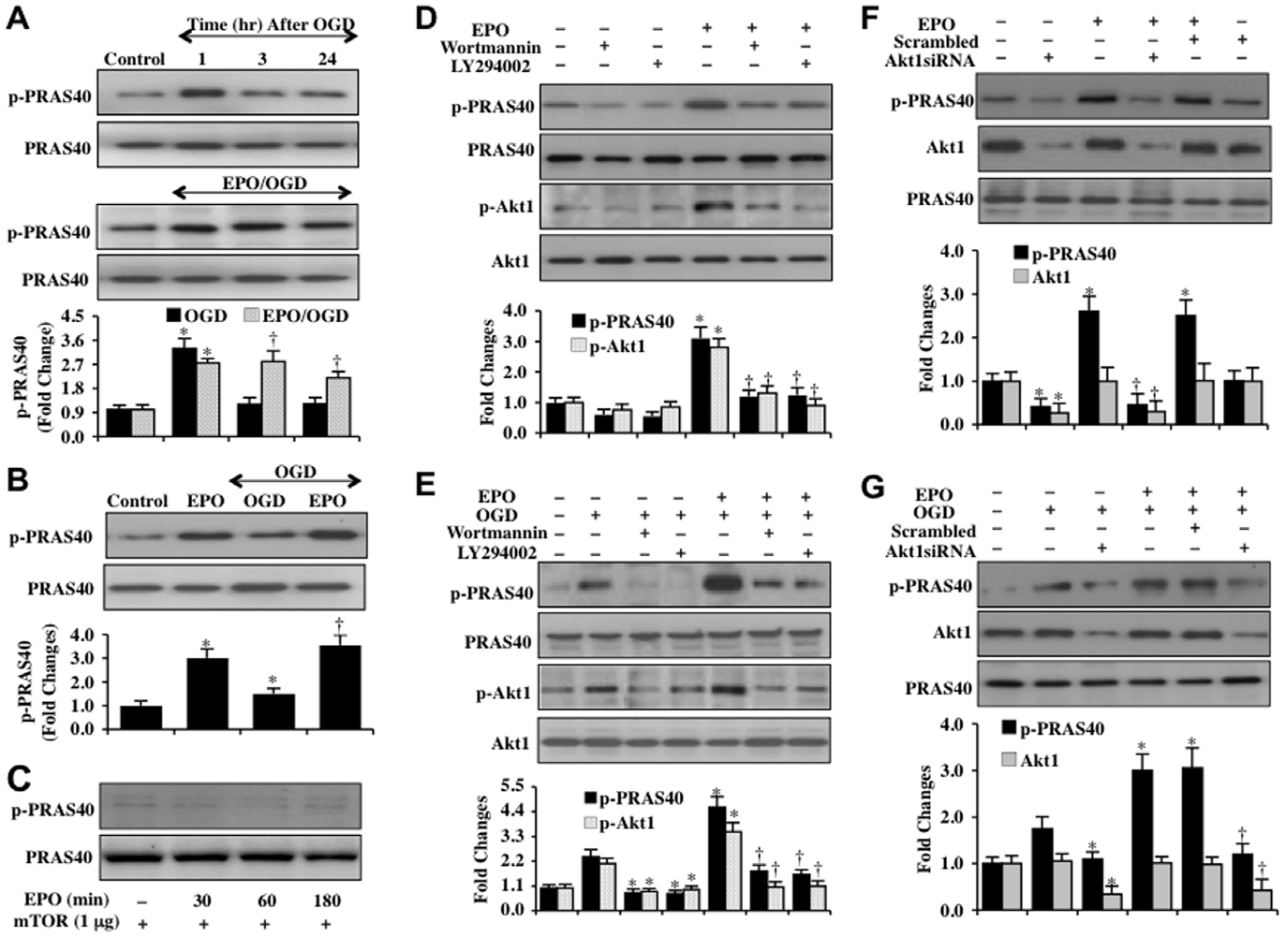
EPO promotes PRAS40 phosphorylation through PI 3-K and Akt during OGD exposure. (**A**) Western blot analysis was performed for phosphorylated (p-)-PRAS40 (p-PRAS40, Thr^246^) in SH-SY5Y cells at 1, 3, or 24 hours (hrs) following OGD exposure. EPO (10 ng/ml) applied to cell cultures 1 hour prior to OGD maintained p-PRAS40 expression at significant levels over 24 hours following OGD (*P<0.01 vs. Control; ^†^ *P*<0.01 vs. OGD at corresponding time points). (**B**) EPO (10 ng/ml) applied to SH-SY5Y cells significantly increased the expression of phosphorylated (p-) p-PRAS40 3 hours later. EPO (10 ng/ml) applied to SH-SY5Y cultures 1 hour prior OGD significantly increased the expression of p-PRAS40 3 hours following OGD (*P<0.01 vs. untreated control; ^†^ *P*<0.01 vs. OGD treated alone). (**C**) EPO (10 ng/ml) was incubated with recombinant PRAS40 protein for 30, 60, and 180 min. No significant expression of phosphorylated (p-) PRAS40 was detected. (**D**) EPO (10 ng/ml) or EPO combined with the P I3-K inhibitors wortmannin (500 nM) or LY294002 (20 μM) were applied to SH-SY5Y cells and western blot analysis for phosphorylated (p-) p-PRAS40 and p-Akt1 (p-Akt1, Ser^473^) was performed 3 hours later. Wortmannin or LY294002 prevented the expression of p-PRAS40 and p-Akt1 during EPO (10 ng/ml) administration (*P<0.01 vs. untreated control; ^†^ *P*<0.01 vs. EPO treated alone). (**E**) EPO (10 ng/ml) was applied to SH-SY5Y cells 1 hour prior to OGD and western blot analysis for phosphorylated (p) p-PRAS40 and p-Akt1 was performed 3 hours following OGD. EPO significantly increased the expression of p-PRAS40 and p-Akt1 during OGD exposure. Wortmannin or LY294002 prevented the phosphorylation of PRAS40 and Akt1 during EPO administration following OGD (*P<0.01 vs. OGD treated alone; ^†^ *P*<0.01 vs. EPO/OGD). (**F**) Transfection of Akt1 siRNA prior to the application of EPO (10 ng/ml) in SH-SY5Y cells significantly limited the expression of Akt1 and significantly reduced the expression of phosphorylated (p-) p-PRAS40 3 hours after administration of EPO. Scrambled siRNA transfection did not alter the expression of Akt1 and p-PRAS40 during EPO application (*P<0.01 vs. untreated control; ^†^ *P*<0.01 vs. EPO treated alone). (**G**) Akt1 siRNA was transfected into SH-SY5Y cells prior to EPO (10 ng/ml) application and OGD exposure. Western analysis expression of phosphorylated (p-) p-PRAS40 and Akt1 was determined 3 hour following OGD. EPO (10 ng/ml) increased p-PRAS40 expression following OGD. Transfection of Akt1 siRNA significantly limited p-PRAS40 expression during OGD alone and during EPO treatment with OGD (*P<0.01 vs. OGD; ^†^ *P*<0.01 vs. EPO/OGD). In all cases above, each data point represents the mean and SD from 3 experiments.

Western blot analysis for phosphorylated p-PRAS40 (Thr^246^) was performed at 1, 3, and 24 hours following OGD exposure. In Figure 4A, phosphorylated p-PRAS40 expression was initially increased within 1 hour following OGD exposure, but over a 24 hour course returned to approximately untreated control levels. In contrast, application of EPO (10 ng/ml) during OGD exposure, phosphorylation of PRAS40 was significantly increased and maintained over a 24 hour course when compared to exposure to OGD alone (Figure 4A). In addition, EPO (10 ng/ml) in normoxic cells not exposed to OGD also significantly increased phosphorylation of PRAS40 within 3 hours of treatment (Figure 4B). To determine whether EPO could directly phosphorylate PRAS40, we incubated recombinant PRAS40 protein with EPO (10 ng/ml) for 30, 60, and 180 minutes in kinase assay buffer and western analysis was subsequently performed. EPO did not directly phosphorylate PRAS40 over a 3 hour time period (Figure 4C).

We next examined the role of the PI 3-K pathway for EPO to phosphorylate PRAS40. EPO (10 ng/ml) was administered without (Figure 4D) and with OGD exposure (Figure 4E) and the expression of phosphorylated p-PRAS40 and phosphorylated Akt1 (p-Akt1) was determined 3 hours later. EPO significantly increased the expression of both p-PRAS40 and p-Akt1 either alone or in the presence of OGD exposure (Figures 4D and 4E). Yet, co-administration of the PI 3-K inhibitors wortmannin (500 nM) or LY294002 (20 μM) prevented EPO from significantly phosphorylating PRAS40 or Akt1 either alone or during OGD exposure (Figures 4D and 4E), illustrating that EPO was dependent upon the PI 3-K pathway to phosphorylate PRAS40.

To assess whether Akt1 played a role during EPO phosphorylation of PRAS40, we examined the effects of gene silencing of *Akt1* on PRAS40 phosphorylation during EPO administration alone and during EPO application with OGD exposure. In Figures 4F and 4G, transfection with Akt1 siRNA significantly prevented the expression of Akt1 during EPO administration alone and during OGD exposure. In addition, gene silencing of *Akt1* eliminated the ability of EPO to phosphorylate PRAS40 without OGD exposure and during OGD exposure, illustrating that EPO also relies upon Akt1 to phosphorylate PRAS40 (Figures 4F and 4G). Non-specific scrambled siRNA did not alter PRAS40 phosphorylation illustrating the specificity for Akt1 in relation to PRAS40 phosphorylation.

### EPO promotes PRAS40 binding to 14-3-3 protein that is PI 3-K dependent

**Figure 5:**
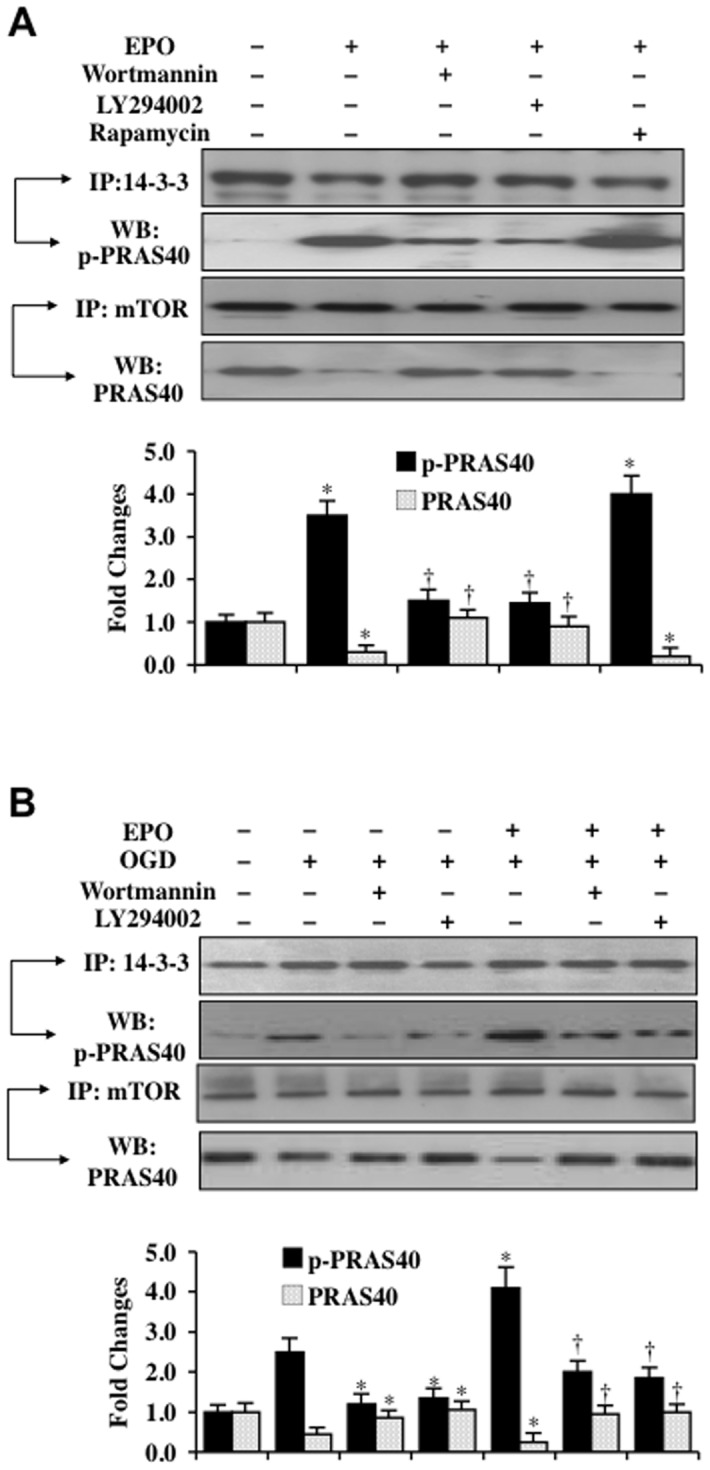
EPO promotes the binding of PRAS40 to protein 14-3-3 through PI 3-K. (**A**) EPO (10 ng/ml) or EPO combined with wortmannin (500 nM), LY294002 (20 μM) or rapamycin (50 nM) was applied to SH-SY5Y cells and cell extracts 3 hours later were immunoprecipitated by antibodies against protein 14-3-3 or mTOR. Western blot analysis was performed to detect the expression phosphorylated (p-) p-PRAS40 and total PRAS40 in the precipitates (*P<0.01 vs. untreated control; ^†^ *P*<0.01 vs. EPO treated alone). Application of EPO increased the expression of p-PRAS40 in the precipitate. In contrast, wortmannin or LY294002 significantly reduced expression of p-PRAS40 in the precipitate. (**B**) EPO (10 ng/ml) or EPO combined with wortmannin (500 nM) or LY294002 (20 μM) was applied to SH-SY5Y cells 1 hour prior to OGD and cell extracts 3 hours following OGD were immunoprecipitated by antibodies against protein 14-3-3 or mTOR. Western blot analysis was performed to detect the expression phosphorylated (p-) p-PRAS40 and total PRAS40 in the precipitates (*P<0.01 vs. OGD; ^†^ *P*<0.01 vs. EPO/OGD). Application of EPO increased the expression of p-PRAS40 in the precipitate during OGD. In contrast, wortmannin or LY294002 significantly reduced expression of p-PRAS40 in the precipitate during OGD. In all cases above, each data point represents the mean and SD from 3 experiments.

The PI 3-K/Akt pathway can phosphorylate threonine^246^ on PRAS40 and result in the dissociation of PRAS40 from the mTOR complex mTORC1 [38]. This ultimately leads to the binding of phosphorylated PRAS40 to the docking protein 14-3-3 to inhibit PRAS40 and activate mTOR signaling [63], [64]. We therefore examined whether EPO alone or during OGD exposure altered the binding of PRAS40 to protein 14-3-3 by immunoprecipitation. EPO (10 ng/ml) significantly increased the expression of p-PRAS40 in the lysate that was immunoprecipitated by antibody against 14-3-3 protein, but decreased the expression of PRAS40 in the lysate that was immunoprecipitated by anti-mTOR the antibody (Figure 5A), suggesting that EPO dissociates PRAS40 from mTOR and increases the binding of p-PRAS40 to protein 14-3-3. Application of EPO with the PI 3-K inhibitors wortmannin (500 nM) or LY294002 (20 μM) reduced the expression of p-PRAS40 in the lysate that was immunoprecipitated by antibody against 14-3-3 protein and increased the expression of PRAS40 in the lysate that was immunoprecipitated by anti-mTOR antibody (Figure 5A), illustrating that EPO relies upon the PI 3-K pathway to foster PRAS40 binding to 14-3-3. Inhibition of the mTOR pathway with application of rapamycin (50 nM) did not alter the ability of EPO to promote PRAS40 binding to protein 14-3-3, further supporting the role of the PI 3-K pathway that is responsible for phosphorylation of PRAS40 (Figures 4D and 4E). In Figure 5B, EPO (10 ng/ml) during OGD exposure also significantly increased the binding of p-PRAS40 to 14-3-3 protein. Furthermore, EPO treatment during OGD with the PI 3-K inhibitors wortmannin (500 nM) or LY294002 (20 μM) limited PRAS40 binding to 14-3-3 protein (Figure 5B), demonstrating that EPO during OGD exposure also relies upon the PI 3-K pathway to promote PRAS40 binding to 14-3-3.

### EPO and gene suppression of PRAS40 limit caspase 3 activation during OGD

**Figure 6:**
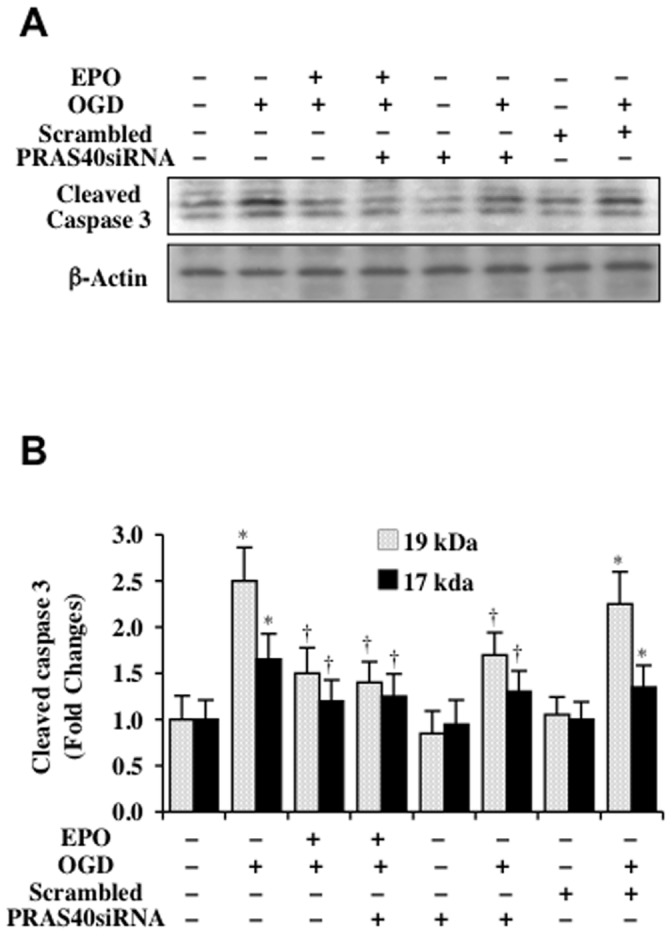
EPO and gene silencing of PRAS40 reduce caspase-3 activation during OGD. (**A**) EPO (10 ng/ml) was applied 1 hour prior to OGD in SH-SY5Y cells. Western blot analysis was performed for the cleaved fragments of caspase 3 with a antibody that identifies both the 19 kDa and 17 kDa fragments of caspase 3 hours following OGD exposure. PRAS40 siRNA transfection significantly reduced the expression of caspase 3 cleaved fragments during OGD exposure and during EPO administration with OGD exposure. (**B**) Quantitative results of the band density of the western blot analysis for caspase 3 fragments show that PRAS40 siRNA transfection significantly reduced the expression of caspase 3 cleaved fragments during OGD exposure and during EPO administration with OGD exposure (*P<0.01 vs. untreated control; ^†^ *P*<0.01 vs. OGD treated alone). Scrambled siRNA transfection did not alter the expression of the caspase 3 fragments during OGD alone or during EPO with OGD. PRAS40 siRNA transfection did not significantly alter caspase 3 fragment expression in untreated cells when compared to cells receiving no treatments. Each data point represents the mean and SD from 3 experiments.

Since EPO can modulate apoptotic cell injury and DNA degradation by limiting caspase activation [17], [21], [30], [52], [65], [66], we assessed whether PRAS40 played a role in this mechanism of cytoprotection by EPO. The expression of cleaved (active) caspase 3 on western analysis was assessed at 6 hours following OGD exposure and demonstrates significant caspase 3 activity (Figures 6A and 6B). Treatment with EPO (10 ng/ml) prevented caspase 3 activation following OGD exposure. In addition, transfection with PRAS40 siRNA significantly decreased caspase 3 activation after OGD exposure and further prevented caspase 3 activation during EPO treatment following OGD exposure (Figures 6A and 6B), suggesting that EPO relies in part on the inhibition of PRAS40 to prevent caspase 3 activation during OGD exposure. Non-specific scrambled siRNA did not alter caspase 3 activity during OGD exposure.

### EPO linked pathways of ERK 1/2 and STAT5 do not alter phosphorylation of PRAS40

**Figure 7:**
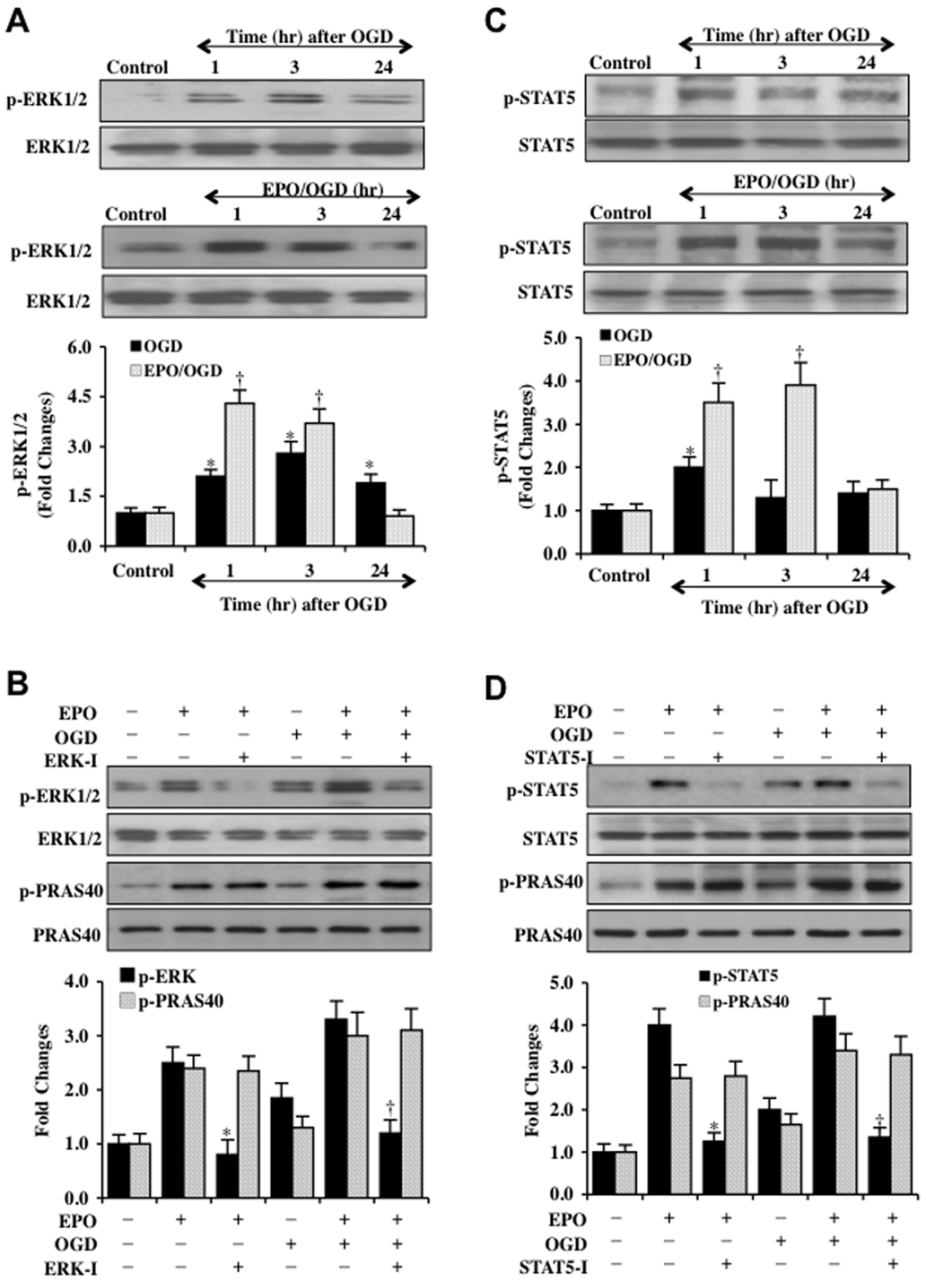
EPO pathways of ERK 1/2 and STAT5 do not alter phosphorylation of PRAS40. (**A**) Western blot analysis for phosphorylated (p-) ERK 1/2 (p-ERK 1/2, Thr^202^/Tyr^204^) in SH-SY5Y cells was performed at 1, 3, or 24 hours (hr) following OGD. EPO (10 ng/ml) that was applied to cell cultures 1 hour prior to OGD significantly increased p-ERK 1/2 expression at 1 and 3 hours following OGD (*P<0.01 vs. Control; ^†^ *P*<0.01 vs. OGD at corresponding time points). (**B**) EPO (10 ng/ml) that was applied to SH-SY5Y cells significantly increased the expression of phosphorylated (p-) p-ERK 1/2 either alone or during OGD exposure 3 hours later. Expression of p-ERK 1/2 was significantly limited during the application of the ERK inhibitor (ERK-I, 100 μM) applied to cultures 30 min prior to EPO administration. Inhibition of ERK 1/2 did not alter the ability of EPO to significantly phosphorylate (p-) p-PRAS40 with or without OGD exposure (*P<0.01 vs. EPO; ^†^ *P*<0.01 vs. EPO/OGD). (**C**) Western blot analysis for phosphorylated (p-) STAT5 (p-STAT5, Tyr^694^) in SH-SY5Y cells was performed at 1, 3, or 24 hours (hr) following OGD. EPO (10 ng/ml) that was applied to cell cultures 1 hour prior to OGD significantly increased p-STAT5 expression at 1 and 3 hours following OGD (*P<0.01 vs. Control; ^†^ *P*<0.01 vs. OGD at corresponding time points). (**D**) EPO (10 ng/ml) that was applied to SH-SY5Y cells significantly increased the expression of phosphorylated (p-) p-STAT5 either alone or during OGD exposure 3 hours later. Expression of p-STAT5 was significantly limited during the application of the STAT5 inhibitor (STAT5-I, 100 μM) applied to cultures 30 min prior to EPO administration. Inhibition of STAT5-I, 100 μM did not alter the ability of EPO to significantly phosphorylate (p-) p-PRAS40 with or without OGD exposure (*P<0.01 vs. EPO; ^†^ *P*<0.01 vs. EPO/OGD). In all cases above, each data point represents the mean and SD from 3 experiments.

Prior studies have demonstrated that EPO can activate extracellular signal related kinase (ERK 1/2) [29], [67], [68] and signal transducer and activator of transcription (STAT5) [29], [69] that may contribute to increased cellular survival during oxidative stress. We therefore investigated whether ERK 1/2 and STAT5 could modulate phosphorylation of PRAS40. Western blot for phosphorylated p-ERK 1/2 (Thr ^202/204^) and phosphorylated p-STAT5 (Tyr^694^) (activate forms of ERK ½ and STAT5) were performed at 1, 3, and 24 hors following OGD exposure. As shown in Figures 7A and 7C, the expression of p-ERK 1/2 and p-STAT5 was initially increased at 1 and 3 hours following OGD exposure. Treatment with EPO (10 ng/ml) further significantly increased the expression of p-ERK 1/2 and p-STAT5 during these time periods following OGD exposure, illustrating that EPO increases the activation of these pathways. In addition, the ability of EPO to increase phosphorylation of ERK 1/2 and STAT5 during OGD exposure was eliminated during application of the ERK inhibitor ERK-I (100 μM) [70] and the STAT5 inhibitor STAT5-I (100 μM) [71] (Figures 7B and 7D). However, application of the ERK inhibitor ERK-I (100 μM) and the STAT5 inhibitor STAT5-I (100 μM) during EPO administration and OGD exposure did not alter the ability of EPO to phosphorylate PRAS40, illustrating that EPO does not rely upon ERK 1/2 or STAT5 to modulate the phosphorylation and activity of PRAS40 (Figures 7B and 7D).

## Discussion

Neurodegenerative disease can lead to disability in multiple systems of the body [72], [73], [74]. In addition, the release of reactive oxygen species during oxidative stress can significantly impact the onset and course of neurodegenerative disorders to influence the outcome of cerebral ischemia [49], [75], [76], neurodevelopment [77], [78], inflammation [79], [80], [81], [82], [83], and cognitive disorders [33], [84], [85]. Novel therapeutic strategies such as EPO may offer great promise to develop new treatments for disease of the nervous system [14], [15], [26], [27], [52], [86], [87]. However, EPO is not without detrimental effects such as during hypertension [88], [89], vascular disease [90], [91], [92], and cancer progression [93], [94], [95], [96]. Therefore it is crucial to identify and target the cellular mechanisms of EPO that can provide robust cytoprotection without detrimental consequences.

We show that treatment with EPO in SH-SY5Y cells prevents cellular injury and apoptotic DNA degradation during exposure to OGD. This cellular protection by EPO is dependent upon the activity of mTOR and its signaling pathways. Prior studies have shown that oxidative stress can block the activity of mTOR signaling pathways to alter cell metabolism and longevity [10], [41], [97] as well as lead to cell death [27], [36], [98]. In contrast, activation of mTOR during oxidative stress can result in cytoprotection [27], [37], [99]. We show that administration of the mTOR inhibitor rapamycin or gene silencing of *mTOR* during EPO application significantly prevented cellular protection by EPO, illustrating that EPO relies upon the activation of mTOR to protect neurons against oxidative stress.

Consistent with prior studies that have demonstrated that EPO increases mTOR signaling in inflammatory cells [27], [33] and in osteoblastic phenotypes in human bone marrow stromal cells [34], we illustrate that EPO in neuronal cells requires mTOR to phosphorylate p70S6K and 4EBP1. Phosphorylation of p70S6K activates this protein and can result in mRNA biogenesis, translation of ribosomal proteins, and cellular proliferation [53], [100]. In regards to 4EBP1, hypophosphorylation of 4EBP1 can block protein translation by allowing 4EBP1 to bind to the eukaryotic translation initiation factor 4 epsilon (eIF4E) through the eukaryotic translation initiation factor 4 gamma (eIF4G), a protein that transfers mRNA to ribosomes. Phosphorylation of 4EBP1 leads to the dissociation of 4EBP1 from eIF4E to allow eIF4G to begin mRNA translation [41], [59]. Prior studies have shown that mTOR depends upon the modulation of p70S6K and 4EBP1 to prevent cell death during apoptosis. Loss of mTOR signaling prevents phosphorylation of both p70S6K and 4EBP1 to lead to apoptosis [101]. In non-neuronal cells of the nervous system, activation of p70S6K by mTOR in astrocytes is cytoprotective through expression of Bcl-2/Bcl-x_L_ expression to block BAD activity that can result in apoptosis [102]. Without significant mTOR activity, 4EBP1 binds to eIF4E that leads to the translation of apoptotic promoting proteins [103].

We also show that phosphorylation of mTOR, p70S6K, and 4EBP1 by EPO are dependent upon the PI 3-K/Akt pathways. Our studies demonstrate that EPO cannot directly phosphorylate mTOR, but requires the PI 3-K/Akt pathways to activate mTOR and phosphorylate p70S6K, and 4EBP1. Prior work has shown that cellular growth and protection can require the involvement of mTOR and the PI 3-K/Akt pathways [60]. The PI 3-K/Akt pathways are principal mediators for cell survival [104], [105], [106], [107], cellular metabolism [108], [109], [110], [111], and tumor progression [112], [113], [114]. EPO also utilizes the PI 3-K/Akt pathway to advance cellular survival in multiple systems of the body [15], [21], [22], [32], [68], [115], [116]. In addition, prior studies suggest that mTOR requires activation of the PI 3-K/Akt pathway to block apoptotic cell death [27], [33], [117], [118]. mTOR also in conjunction with the PI 3-K/Akt pathways can inactivate “pro-apoptotic” forkhead transcription factors to block cellular injury [97], [119], [120].

Given that mTOR signaling through p70S6K and 4EBP1 forms an important component for EPO neuronal protection during oxidative stress, we examined the role of PRAS40 that blocks mTOR activity and prevents the binding of p70S6K and 4EBP1 to Raptor [38], [39], [40]. We show that gene silencing of *PRAS40* prevents both cellular injury and neuronal apoptosis during oxidative stress. Furthermore, gene silencing of *PRAS40* during EPO administration and OGD exposure enhanced cell survival and further reduced genomic DNA degradation to a greater extent than EPO alone, suggesting that EPO relies upon PRAS40 inhibition for cellular protection. This increased degree of protection by EPO during the loss of PRAS40 appears to be tied to the promotion of mTOR signaling for EPO, since gene silencing of *PRAS40* increased the phosphorylation of p-mTOR, p-p70S6K, and p-4EBP1 during EPO treatment. EPO also maintained the phosphorylation and inhibition of PRAS40 over a 24 hour course following OGD exposure to a significantly greater extent that during exposure to OGD alone. In other cell systems, phosphorylation of PRAS40 has resulted in decreased apoptotic cell death [42], [43], [44]. Loss of PRAS40 through gene silencing in HeLa cells also has been shown to prevent apoptosis against tumor necrosis factor and cyclohexamide [121].

EPO controls PRAS40 activity through the post-translational phosphorylation of PRAS40 and the subcellular binding of PRAS40 to protein 14-3-3. However, EPO does not appear to directly control the post-translational phosphorylation of PRAS40, but requires activation of the PI 3-K/Akt pathway similar to the regulation of mTOR by EPO. We show that *in vitro* incubation of EPO with PRAS40 does not lead to phosphorylation of PRAS40. In contrast, co-administration of the PI 3-K inhibitors wortmannin or LY294002 or gene silencing of *Akt1* eliminated the ability of EPO to phosphorylate PRAS40, suggesting that EPO was dependent upon the PI 3-K/Akt pathways to phosphorylate PRAS40. Akt can phosphorylate threonine^246^ on PRAS40. This results in the dissociation of PRAS40 from mTORC1 [38] and the binding of PRAS40 to protein 14-3-3 to allow activation of mTOR signaling [63], [64]. Consistent with these studies for PRAS40, we demonstrate that EPO also fosters the binding of phosphorylated PRAS40 to protein 14-3-3. Application of EPO with the PI 3-K inhibitors wortmannin or LY294002 significantly prevented phosphorylated PRAS40 binding to protein 14-3-3, illustrating that EPO also employed the PI 3-K pathway to compartmentalize PRAS40 in the cell with protein 14-3-3. Yet, treatment with rapamycin did not affect phosphorylated PRAS40 binding to protein 14-3-3 during EPO administration, further demonstrating that control of phosphorylated PRAS40 binding to protein 14-3-3 by was modulated at the level of the PI 3-K pathway.

Control of neuronal apoptosis by EPO involves suppression of caspase 3 activation through PRAS40 but not through ERK 1/2 or STAT5 pathways. Both the early and late phases of apoptotic cell injury can be the result of caspase 3 activation [4], [113], [122], [123], [124], [125] and EPO has been shown to effectively control caspase activity [15], [21], [30], [50], [66], [126], [127]. We show that loss of PRAS40 during transfection with PRAS40 siRNA significantly limits caspase 3 activation after OGD exposure. In addition, caspase 3 activation following OGD exposure is decreased to a greater degree by EPO during gene silencing of PRAS40 than with EPO alone, suggesting that PRAS40 controls caspase activation and mediates the ability of EPO to block caspase 3 activity. EPO also uses the pathways of ERK 1/2 and STAT5 to prevent apoptotic cell injury [29], [67], [68]. Yet, we found that phosphorylation of PRAS40 by EPO was not associated with ERK 1/2 and STAT5, suggesting that ERK 1/2, STAT5, and PRAS40 represent independent cytoprotective pathways for EPO.

Our studies offer new insight into the neuroprotective pathways for EPO during OGD that can lead to oxidative stress. EPO prevents cell injury through activation of mTOR signaling and is dependent upon the inhibition of PRAS40 as vital regulatory pathway. EPO controls the mTOR signaling pathways of p70S6K, 4EBP1, and PRAS40 through PI 3-K and Akt. PRAS40 either alone or as a component of EPO signal transduction governs cell survival through caspase 3 activation that is independent of other cytoprotective pathways of EPO that involve ERK 1/2 and STAT5. Future studies that can further elucidate the role of PRAS40 and the ability of EPO to regulate this pathway may offer novel approaches for the treatment of a variety of multi-system disorders, such as those that involve neurodegeneration.
